# Ligament-Focused Advanced Monopolar Radiofrequency: A New Approach to Face and Neck Skin Laxity

**DOI:** 10.7759/cureus.109867

**Published:** 2026-05-29

**Authors:** Daniel Dziabas

**Affiliations:** 1 Dermatology, Dziabas Academy and Research Institute, São Paulo, BRA

**Keywords:** face lift, ligaments, neck, noninvasive monopolar radiofrequency, skin laxity

## Abstract

Facial and neck skin laxity due to aging represents a major aesthetic concern among women. Despite this, most patients prefer non-invasive treatments that may offer natural results, minimal downtime, and low risk of complications. Advanced monopolar radiofrequency (RF) technology addresses these needs through continuous low-temperature epidermal cooling, enabling safe delivery of maximal energy (140 W) to superficial and mid-dermal layers and facial retaining ligaments without burns, lesions, or discomfort. This protocol presented a novel ligament-focused approach for adults aged 35 years or older with mild-to-moderate facial and/or neck skin sagging. Applying 1,000 shots in one session across facial, submental, and neck regions, targeting ligaments and fibrous septa, the protocol was delivered in a hashtag pattern with 50% overlap at energy levels 8-10. Clinical observations demonstrated an improvement in skin thickness, texture, elasticity, facial and neck contour, and wrinkle reduction, achieving lifting effects with minimal/no pain and high patient satisfaction. Reapplication is recommended after six months to optimize and maintain the results and, for moderate and severe cases, we recommend a combination of new monopolar RF with fillers or microfocused ultrasound. In conclusion, the protocol offers versatile skin rejuvenation by promoting collagen remodeling and contraction of fibrous septa and ligaments, providing a good alternative treatment for facial aging with an interesting balance between results and comfort.

## Introduction

Aging is a real concern among all adults, and treatments that decrease its effects are increasing worldwide [1]. Despite this, according to the Global Data, 76% of people prefer treatments that improve their appearance gradually, with natural results, rather than drastic or "artificial" changes [2].

To achieve these results, patients seek painless procedures, shorter downtime, and minimal risk of injuries or complications. Monopolar radiofrequency (RF) technology is one alternative for these aesthetic objectives, reducing and preventing facial and cervical skin laxity. It is a non-invasive and non-ablative procedure [3,4] that utilizes high-frequency alternating electrical current (typically 0.3-10 MHz) passed between electrodes to generate controlled thermal energy within dermal and subcutaneous tissues [5]. The heating caused by RF--temperatures between 50 and 60ºC--can interact with the cellular components of the skin, partially denaturing the proteins and stiffening their structures [6,7], leading to the contraction of collagen fibers and reducing skin sagging with a skin-tightening effect [8,9].

Especially the fibrous septa, connective tissue bands rich in collagen and elastin that cross from the dermis and the subcutaneous layer, are responsible for partitioning fat into lobules and anchoring the skin to deeper layers. This low-impedance network that surrounds fat conducts the energy applied to it, improving the tightness of the skin and, consequently, the contour of the face [10,11]. The network of facial retaining ligaments, another fibrous connective tissue structure that includes the zygomatic, mandibular, masseteric, and orbicularis retaining ligaments, also contributes to anchoring the skin and superficial musculoaponeurotic system (SMAS) to deeper bony and fascial planes [11]. With aging, these ligaments weaken, leading to the descent of facial fat and soft tissues, contributing to sagging and the formation of jowls and deep nasolabial folds [12,13]. 

A special, new, advanced monopolar RF technology delivers a more precise energy to deeper structures, including retaining ligaments, with continuous, and very low-temperature cooling, allowing the application of maximal energy (up to 140 W) to the skin without risk of burning, minimizing patient discomfort and ensuring high-power treatment delivery. Consequently, a technique focused on facial retaining ligament treatment that targets skin layers and fibrous septa without risking nerve or ligament damage could optimize patient outcomes in skin tightening and lifting effects, potentially offering more comprehensive facial rejuvenation [5,14]. This technological capability could lead to protocols that systematically target deeper structures, including facial retaining ligaments, with appropriate safety margins.

Despite the extensive literature on RF for facial rejuvenation, existing protocols do not specifically target facial retaining ligaments and focus primarily on generalized dermal heating rather than anatomically directed treatment of specific supporting structures. Therefore, the objective of this paper is to demonstrate a new technique focused on ligament treatment using a new advanced monopolar RF for improving skin laxity in the face and neck.

## Technical report

Indication

Women and men, more than 35 years old, who complain about mild or moderate sagging in facial and/or neck areas. Patients with recent facial procedures or surgery (<3 months), facial nerve injury, significant sun damage or photoaging, autoimmune connective tissue disorders, active local infection, pregnancy, skin disorders such as keratitis, recent scars, severe acne, psoriasis, acute inflammatory facial dermatoses such as rosacea, patients undergoing photosensitizing treatment, bleeding disorders, oral anticoagulant use, or implanted electronic devices such as pacemakers are contraindicated from the procedure.

Treatment protocol

Before the procedure, the skin should be cleansed and shaved, if needed. The ground pad of the Monopolar RF (Coolfase™, Asterasys Co., Seoul, Republic of Korea) should be placed on a distant anatomical site to complete the electrical circuit and facilitate electromagnetic field generation. This pad is generally placed on the patient’s back, avoiding bony structures and hairy areas to ensure optimal contact and safety.

The technique delivers 1,000 total shots per patient, distributed across the face, submental region, and neck. Energy is delivered at levels ranging from 8 to 10 (119-133J--depending on the patient's sensibility), with a 50% overlap in application. On the face and submental region, two RF phases are applied: the first on the retaining ligaments and the second on the total area. On the neck, only one phase is applied.

**Figure 1:**
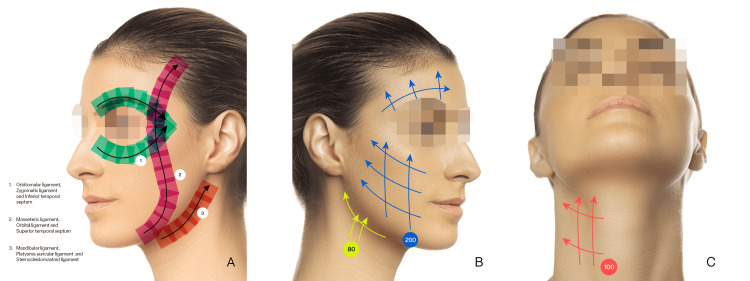
(A) Facial ligaments that will receive targeted energy (phase 1-greenlines (1): orbitomalar and zygomatic ligament and inferior temporal septum ligaments. Pink lines (2): masseteric and orbital ligaments and superior temporal septum. Red lines (3): mandibular, platysma auricular, and sternocleidomastoid ligaments). (B) and (C) Schematic points of application and the number of shots over the full face and anterolateral neck. (A) The ligaments observed in photo A were created by Georgia Caroline de Oliveira from Entera using Adobe Illustrator to create the vectorial graphic images. No in-built AI tools were used or any other generative AI tools were used to create figure (A). (B) and (C) Written consent was obtained from the patient for publication in an open-access journal.

In the facial region, 300 shots are delivered per hemiface. In the first phase, 100 shots per hemiface are applied over the facial retaining ligaments. In the temporal septa, 20 shots are applied along the temporal region, targeting fibrous attachments. In the orbital retaining ligament, 20 shots are applied along the inferior orbital rim, avoiding the direct orbital area. In the zygomatic retaining ligament, 20 shots, over the zygomatic prominence; masseteric ligament, 20 shots along the anterior border of the masseter muscle, and mandibular ligament, 20 shots along the mandibular border (Figure 1A).

In phase 2, two complete passes over the full facial (100 shots per pass), covering the forehead, temple, periorbital (avoiding direct orbital area), cheek, perioral, and jaw regions in a hashtag (#) pattern with 50% overlap (Figure 1B).

In the submental region, 100 shots are delivered per side. In phase 1, 20 shots above the hyoid-related ligaments are delivered along the stylohyoid attachment region; 10 shots on the mylohyoid and 10 shots on the stylohyoid ligaments, and, in phase 2, 80 shots are distributed over two passes (40 shots per pass) in a hashtag configuration with 50% overlap, covering from the mental protuberance to the hyoid bone (Figure 1B).

In the neck region, a total of 100 shots is applied per side, delivered in two passes (50 shots per pass) using a hashtag pattern (Figure 1C), covering the anterolateral neck from the mandibular border to the clavicle, avoiding central anterior structures such as the trachea.

Mild erythema is expected as a positive predictor of effective energy delivery, and edema may be present for 24-48 hours. Broad-spectrum sunscreen (SPF ≥30) should be applied immediately after the procedure and daily thereafter, and patients are advised to avoid sun exposure. Patients should maintain daily gentle cleansing and moisturizing, avoid vigorous exercise for 24 hours, and avoid facial treatments for at least two weeks.

## Discussion

**Figure 2:**
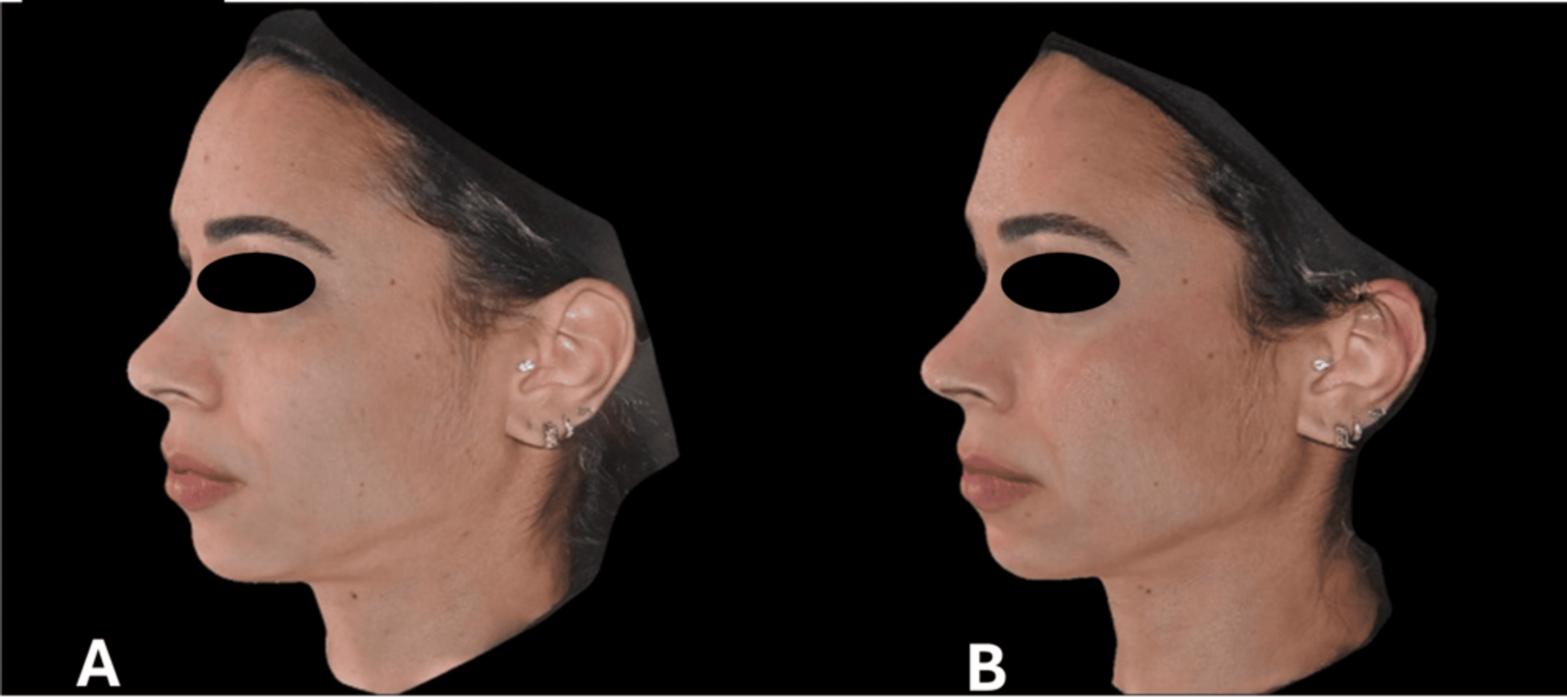
Example of a woman who applied the technique. (A) Pre-procedure and (B) post-procedure. A lifting effect of the eyebrows and an improvement in mandibular contour are observed, comparing the left to the right photo. Written consent was obtained from the patient for publication in an open-access journal.

According to our clinical experience, after only one session, it is possible to observe an improvement in skin thickness, texture, and elasticity, and consequently, in skin sagging. Moreover, there is an improvement mainly in the middle and lower face, including jawline contour and a reduction in wrinkles, leading to a global aesthetic improvement, with mild or no pain during the procedure and high patient aesthetic satisfaction, as observed in this example (Figure 2).

Several minimally invasive or non-invasive procedures have been developed for skin aging. Among them, a new advanced monopolar RF technology has demonstrated significant improvement in skin laxity, providing an important lifting effect, minimal downtime, and generally no or mild adverse effects. This technique can be used as a stand-alone treatment in cases of mild sagging [5,15,16] or for those who seek prejuvenation, or it can be combined with other energy-based devices [14,17] or injectable fillers [14,18] in cases of moderate to severe sagging.

Radiofrequency differs from other energy-based devices [10,17], especially with the advancements in the latest monopolar RF technology. Unlike microfocused ultrasound (MFU), which primarily targets deeper tissue layers, RF mainly affects the superficial to mid-dermal layers [19]. MFU delivers focused thermal coagulation points at precise depths, leading to collagen denaturation, neocollagenesis, and coagulative necrosis of adipocytes, increasing the risks of thermal injury [20]. In contrast, this new technology RF releases continuous epidermal cooling, enabling the safe delivery of up to 140 W of power without causing discomfort and/or tissue lesions, providing an optimal balance between efficacy, comfort, and safety.

The subcutaneous tissue is composed of fibrous septa that form a connective tissue network surrounding the adipose tissue. Fat acts as a thermal insulator and does not effectively propagate heat. In contrast, collagenous septa, which have low impedance, readily conduct energy and consequently, contract the collagen when heated. RF induces dermal remodeling primarily through collagen denaturation and neocollagenesis [16] and may be particularly interesting in patients with facial volume changes following weight loss or in post-menopausal women, as RF does not induce compaction of adipose tissue [15].

Another interesting aspect of this technique, when applied with the new monopolar RF, is the ability to target facial retaining ligaments safely. These fibrous connective structures, such as zygomatic, mandibular, masseteric, and orbicularis, anchor the skin and superficial tissues to deeper planes and help the skin tighten and lift effects [13]. As facial ligaments weaken with age, they fail to support overlying fat pads and soft tissues, resulting in gravitational descent that manifests as jowls and deepened nasolabial folds [12]. Controlled heating of these structures induces collagen contraction and shrinkage of the fibrous tissue, including ligaments [15]. The integrated system of continuous cooling enables us to apply energy to the ligaments with minimal risk of nerve injury compared with other devices, reaching excellent outcomes, such as skin tightening, dermal remodeling, and wrinkle reduction.

Our clinical experience has observed that better and long-term results are achieved when the procedure is reapplied after six months. Another observation is that in cases of mild facial sagging, this technique is sufficient to achieve the “lifting effect” and maintain it in the long term. In cases of moderate or severe facial sagging, the association with injectable fillers and/or MFU should be considered after RF, with very interesting results and high patient satisfaction.

Although the lack of a comparative clinical study with systematic data collection is a potential limitation, the clinical results reported in this paper may be validated in future prospective controlled trials.

## Conclusions

This preliminary description of the results of a new advanced monopolar RF technique targeting facial and neck ligaments suggested potential benefits as a versatile approach to skin rejuvenation. By delivering controlled energy to the superficial to mid-dermal layers and the supporting ligamentous structures, the protocol appears to stimulate collagen remodeling and may promote progressive tightening and lifting effects.

This technique is potentially well-suited to improving skin laxity and enhancing facial contour definition in a minimally invasive way with a favorable balance between efficacy and precision. However, as a preliminary study relying on subjective outcomes, larger controlled trials with objective measures are essential to confirm these observations and establish long-term efficacy.
